# Enhancing Nutraceutical Efficacy: The Role of M.A.T.R.I.S. Technology in Modulating Intestinal Release of Lipoic Acid and L-Carnitine

**DOI:** 10.3390/ijms26104866

**Published:** 2025-05-19

**Authors:** Rebecca Galla, Sara Ferrari, Simone Mulè, Marino Nebuloni, Marco Calvi, Mattia Botta, Francesca Uberti

**Affiliations:** 1 Noivita S.r.l.s., Spin Off of University of Piemonte Orientale, Via Solaroli 17, 28100 Novara, Italy; rebecca.galla@uniupo.it; 2 Laboratory of Physiology, Department for Sustainable Development and Ecological Transition, University of Piemonte Orientale, UPO, 13100 Vercelli, Italy; sara.ferrari@uniupo.it (S.F.); simone.mule@uniupo.it (S.M.); 20035008@studenti.uniupo.it (M.B.); 3 Redox Srl, Viale Stucchi 62/26, 20900 Monza, Italy; marino.nebuloni@fastwebnet.it (M.N.); solidstate@labredox.com (M.C.)

**Keywords:** delivery system, controlled release, intestinal absorption, nutraceuticals

## Abstract

A major challenge in developing new functional foods is effectively protecting and releasing bioactive compounds in specific body areas. The Multiform Administration Timed Release Ingredients System (M.A.T.R.I.S.) is an innovative method that coats active ingredient particles with a permeable membrane, allowing for diffusion without the presence of inactive materials. This study aimed to test how M. A. T. R. I. S. modulated the absorption and effects of two molecules: α-lipoic acid and acetyl-L-carnitine. This study examined the structures of these molecules with or without M.A.T.R.I.S. and investigated their intestinal absorption. Peripheral nervous system analyses were also conducted to confirm the ability of substances to maintain their functions in the presence of M.A.T.R.I.S. Results showed that M.A.T.R.I.S. modulated the absorption of both molecules compared to granular raw material forms (*p* < 0.05). Additionally, the M.A.T.R.I.S. molecules better supported peripheral nerve well-being than their granular raw material forms (*p* < 0.05). In conclusion, this study demonstrates that M.A.T.R.I.S. technology can be used to create innovative, safe treatments by enhancing absorption mechanisms to improve the effectiveness of substances in reaching their specific targets.

## 1. Introduction

A significant challenge for researching and developing novel functional foods is designing and creating new strategies for protecting bioactive compounds and their distribution to specific sites. Many bioactive compounds exhibit low water solubility, low cellular uptake, and reduced efficacy due to nonspecific biodistribution and rapid elimination [1]. To design a release distribution and rate strategy, it is important to know the fundamental rules of controlled release procedures; indeed, the release process can be time-, site-, rate-, and stimulus-specific, which can be established by one or a combination of different release mechanisms such as degradation, diffusion, erosion, dissolution, swelling, osmosis, and fragmentation [2,3]. However, there are also various release mechanisms: the sustained one, which refers to the process of constant release over time; the burst mechanism, which implies the rapid release of bioactive in a short period; the targeted one, which alludes to the release of bioactive principle in a specific site of the human body that can be established through the use of engineered nanoparticles with the potential to adhere to a specific biological surface through the gastrointestinal tract to improve the efficiency of nano-delivery systems; and finally the triggered one, which indicates the burst release of bioactive encapsulated in designed nano-delivery systems that can respond to changes in defined environmental triggers [2,4].

For several years, there have been different types of controlled release systems that have been developed with the aim of modulating drug release. One of the first release systems introduced is the Spansule technology, which is able to control the dissolution of the drug’s nucleus through a coating that protects the molecule from gastrointestinal fluids and allows for its controlled dissolution. More generally, controlled dissolution and diffusion systems dominate the number of Food and Drug Administration (FDA)-approved formulations [5]. In diffusion-controlled release systems, drugs are trapped and released by diffusion through water-insoluble inert polymer membranes (tank systems) or polymeric matrices (monolithic systems) [6]. These polymer membranes are used throughout the pharmaceutical industry as carriers for the drug, representing a supporting structure in conventional and controlled-release formulations. Polymers that can be used to create these sustained-release systems include hydrophilic, non-cellulosic, and hydrophobic polymers [7]. In this context of diffusion-controlled release systems is the Multiform Administration Timed Release Ingredients System (M.A.T.R.I.S.), a technology based on coating each particle of active ingredient, ranging in size from 200 to 800 μ, with an insoluble and permeable membrane, which acts by diffusion, without any use of other inactive materials. The technology under discussion represents the state of the art in orally sustained-release active ingredients. It features a homogeneous and maximum dispersion of the active ingredient over the entire area of the gastrointestinal tract, ensuring uniform absorption during the established period. It also features a continuous release of the active ingredient by diffusion, calibrated over 8–10 h. This technology has been developed to mitigate the risk of local irritation and undesirable side effects while also offering the possibility of gastro-resistant dosage forms and various forms of administration, including dispersible and oro-soluble single-dose sachets, single-dose vials with dispensing caps, highly soluble tablets, capsules, and tablets in the traditional form [8]. This study aimed to test and refine the M.A.T.R.I.S. technology using two active ingredients with known biological efficacy but with poor bioabsorption properties, such as α-lipoic acid (ALA) and L-carnitine (CAR).

ALA is a compound found in various foods, including meat, fruits, and vegetables, except spinach and broccoli, which contain levels of ALA comparable to those found in animal tissues. ALA is classified as a 1,2-dithiolane-3-pentanoic acid, featuring an asymmetric carbon atom and the ability to exist in both R and S forms [9]. Although ALA is present in these nutritional sources, it is unlikely that sufficient amounts will be consumed. Furthermore, although ALA synthesis occurs in the heart, liver, and testes, the production is low, and only a negligible amount of ALA reaches the bloodstream. Consequently, dietary supplements are typically the primary sources of ALA, and most of the available information regarding its bioavailability pertains to studies employing supplements that attain potentially therapeutic levels [10,11]. During the absorption of ALA, a sodium-dependent multivitamin transporter, on which ALA acts as a substrate, intervenes, contributing to gastrointestinal absorption and tissue transport from plasma [12], resulting in the absorption of 30–40% of an oral dose [13]. In summary, although ALA can offer many benefits to human health, its therapeutic efficacy is constrained by pharmacokinetic limitations. These limitations can be attributed to its poor stability, low bioavailability, and hepatic degradation, which results in a brief half-life [14].

Concerning CAR, it is an amino acid derivative that can be found in most mammals. It has been reported to have many biological functions, and it is specifically involved in helping move long-chain fatty acids from the cytosol into the mitochondria after β-oxidation [15]. This molecule is water-soluble, and since endogenous biosynthesis only produces a limited quantity, food and renal absorption are responsible for maintaining CAR homeostasis [16]. Although carnitine is categorised as a “conditionally essential” nutrient, its low bioavailability and absorption, rapid renal clearance, and active tissue absorption limit the levels of CAR that can be raised in plasma with oral therapy [17].

Based on these premises, this study was designed to confirm the ability of the M.A.T.R.I.S. technology to modulate the pharmacodynamic profile of ALA and CAR, chosen as an application example. In particular, work was conducted on the modulation of ALA and CAR absorption from the intestinal tract and the maintenance of their effects at the level of a final target, such as peripheral neurons. This study also briefly characterised the chemical and morphological structure of the technology.

## 2. Results

This research has been meticulously designed with the overall aim of systematically evaluating the efficacy of the innovative M.A.T.R.I.S. technology in the complex coating process of the chosen molecules, simultaneously modulating, in a controlled manner, the release profiles of these molecules. To achieve this ambitious goal, the technology has been rigorously tested on two distinct molecular compounds, ALA and CAR, to ascertain its performance and applicability to chemical entities of different structures. However, it should be noted that to ensure clarity and comprehensibility in the data presentation, all experimental results on the CAR molecule have been systematically compiled and included in the Appendix A as additional results for reference and further analysis.

### 2.1. Chemical and Morphological Characterisation of α-Lipoic Acid Two Forms

**Figure 1:**
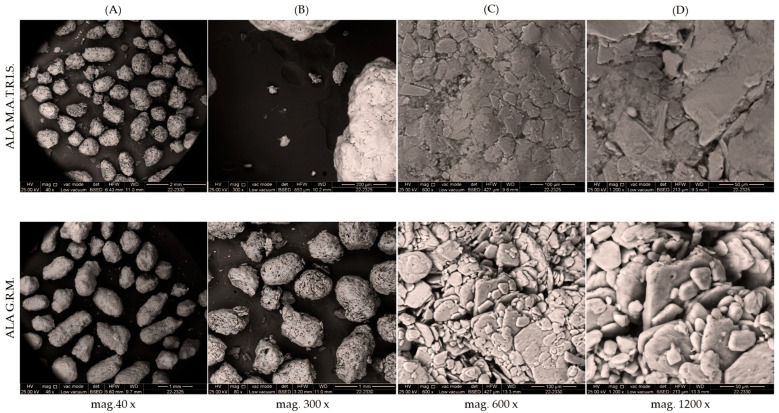
Scanning electron microscopy analysis of two forms of ALA at different magnifications. In (**A**), ALA M.A.T.R.I.S. and ALA G.R.M. at 40× magnification; in (**B**), ALA M.A.T.R.I.S. and ALA G.R.M. at 300× magnification; in (**C**), ALA M.A.T.R.I.S. and ALA G.R.M. at 600× magnification; and in (**D**), ALA M.A.T.R.I.S. and ALA G.R.M. at 1200× magnification. Determinations of particle size were made using SEM images, which were acquired at varying magnifications. These images were then subjected to micrometric measurements. The red line indicates the reference particle size measurement.

**Figure A1:**
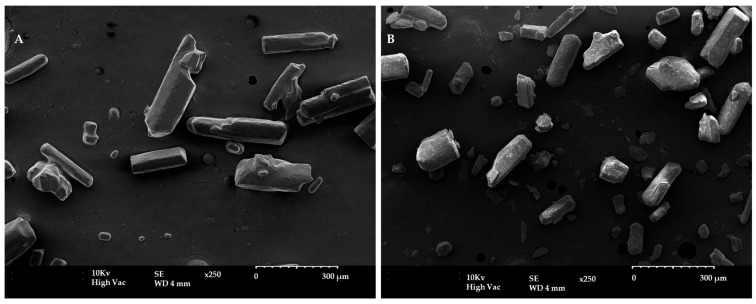
Scanning electron microscopy analysis of two forms of CAR. In (**A**), CAR R.M.G. and in (**B**), CAR M.A.T.R.I.S. powders. Determinations of particle size were made using SEM images acquired at varying magnifications. These images were then subjected to micrometric measurements. The images reported are examples of the results obtained.

To evaluate the potential of M.A.T.R.I.S. technology in enhancing the biological effect of ALA, M.A.T.R.I.S. technology containing ALA vs. ALA Granular Raw Material (G.R.M.) was tested. The aim was to highlight morphological and chemical differences between the two forms of ALA. As shown in Figure 1, the ALA Granular Raw Material powder exhibited a distribution that was essentially homogeneous with granules of similar spherical shape and size, ranging from 500 microns to approximately 1.5 mm. In addition, the presence of microcrystals measuring less than 300 microns was observed in minute amounts. In contrast, the ALA M.A.T.R.I.S. powder exhibited irregularly shaped granules observable under the microscope, with sizes ranging from 500 microns to approximately 1.5 mm. In this sample, too, the presence of microcrystals was observed in small amounts, with a size of less than 100 microns. Based on the observed images obtained by scanning electron microscopy (SEM), it can be defined that the ALA M.A.T.R.I.S. formulation looks very similar to the ALA G.R.M. Similar to what was observed for ALA, SEM morphological investigations of the two forms of CAR (Figure A1 in Appendix A) also showed that the crystals present in the raw material have a parallelepiped shape, with sizes ranging from 70 to 600 microns. In addition, the images showed the absence of irregular microcrystals on the surface and the presence of visible paired (twinned) crystals, typical during the crystallisation process. In contrast, as concerns CAR treated with M.A.T.R.I.S. technology, the images obtained by SEM revealed that the morphology remains largely parallelepipedal, but the size of the crystals is smaller, less than 500 microns. The analysis revealed the presence of irregularly shaped microcrystals (smaller than 100 microns) adhered to the larger parallelepipedal crystals and paired (twinned) crystals, consistent with the raw material. In conclusion, SEM images and reports show that M.A.T.R.I.S. technology successfully modified the surface of CAR crystals. The adherence of irregular microcrystals on the larger crystals suggests that the M.A.T.R.I.S. coating has been effectively applied and can serve various purposes, such as protecting the active ingredient from environmental factors or improving its bioavailability.

**Figure 2:**
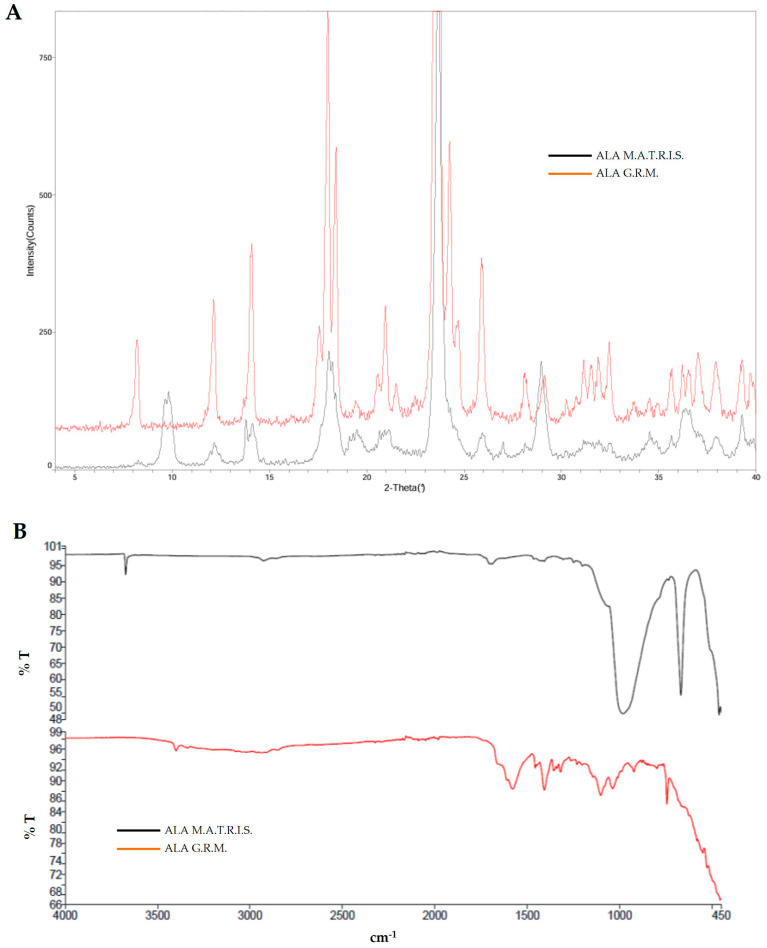
Spectral analyses of two forms of ALA. In (**A**), X-ray diffraction of ALA G.R.M. (red line) and ALA M.A.T.R.I.S. (black line); in (**B**), FT-IR/ATR spectroscopy of ALA G.R.M. (red line) and ALA MATRIS (black line). The images reported are an example of the experiments performed.

Following SEM analysis, X-ray diffraction and Fourier transform infrared spectroscopy (FT-IR)/attenuated total reflection (ATR) spectroscopy analyses were performed. As shown in Figure 2A, the diffraction peaks of ALA G.R.M. and ALA M.A.T.R.I.S. were compared, and it was observed that ALA G.R.M. peaks were also present in the ALA M.A.T.R.I.S. sample. In addition, the uncommon peaks were ascribed to the particle coating film present in the ALA M.A.T.R.I.S. sample. Similar to what was observed from the data obtained from the X-RD analysis, the data obtained from the FT-IR/ATR analysis also showed that the IR bands of ALA G.R.M. are identifiable in the ALA M.A.T.R.I.S. sample profile (Figure 2B). In conclusion, the difference between the two forms of ALA corresponding to the uncommon peaks could also be attributable to the technology used.

**Figure 3:**
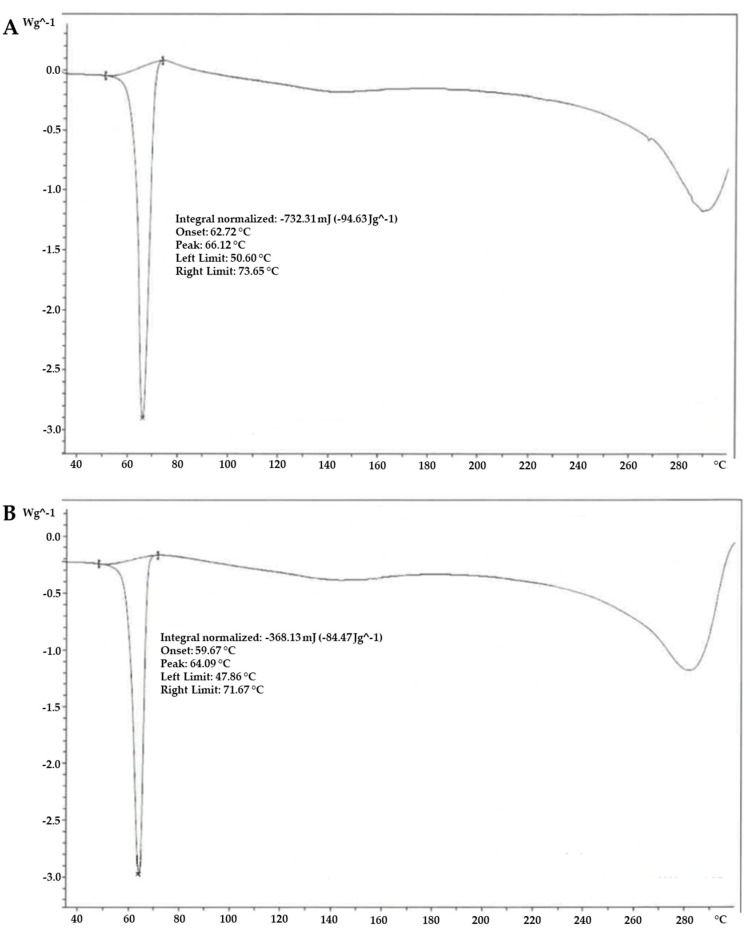
DSC thermal analysis of two forms of ALA. (**A**) ALA G.R.M. and (**B**) ALA M.A.T.R.I.S. The images reported are an example of the results obtained.

To conduct comprehensive chemical and morphological assessments of the two ALA forms under investigation, Differential Scanning Calorimetry (DSC) was utilised as a thermal analysis method. As illustrated in Figure 3, the DSC analysis revealed a melting endotherm for the ALA M.A.T.R.I.S. sample within the temperature range of 48–72 °C (with an onset at 60 °C; enthalpy: −85 J/g), which can be attributed to the ALA G.R.M., exhibiting a melting endotherm spanning 51–74 °C (with an onset at 63 °C; enthalpy: −95 J/g). In addition, it was observed from the DSC thermal analysis that the melting temperature of ALA was at about 60 °C (onset) for both forms of ALA under study (G.R.M. and M.A.T.R.I.S.), with a reduced number of endothermic events attributable to the M.A.T.R.I.S. technology used.

### 2.2. Intestinal Health Analysis After ALA Treatment

**Figure 4:**
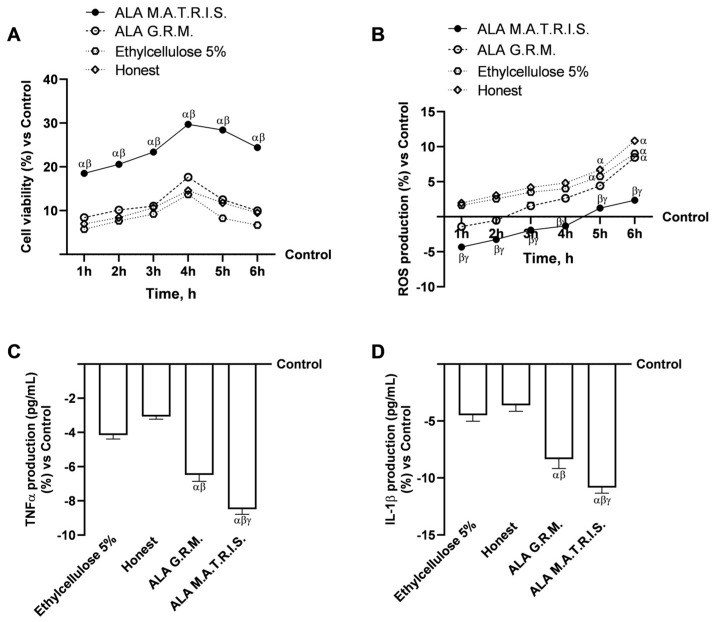
Intestinal health analysis after stimulation with ALA forms. In (**A**), cell viability was measured by MTT test; in (**B**), ROS production was evaluated by Cytochrome C reduction; in (**C**), TNFα, and in (**D**), IL-1β production were both measured by specific Enzyme-Linked Immunosorbent Assay (ELISA) Kit. ALA = α-Lipoic Acid at 50 µM. Data are expressed as mean ± SD (%) of 5 independent experiments, each performed in triplicate; experiments normalised to the control (0%) line. For MTT analysis: *p* < 0.05 vs. Control; α *p* < 0.05 vs. ALA G.R.M.; β *p* < 0.05 vs. excipients (Ethylcellulose 5% and Honest). For ROS, TNFα, and IL-1β analysis: α *p* < 0.05 vs. Control; β *p* < 0.05 vs. ALA G.R.M.; γ *p* < 0.05 vs. excipients (Ethylcellulose 5% and Honest).

**Figure 5:**
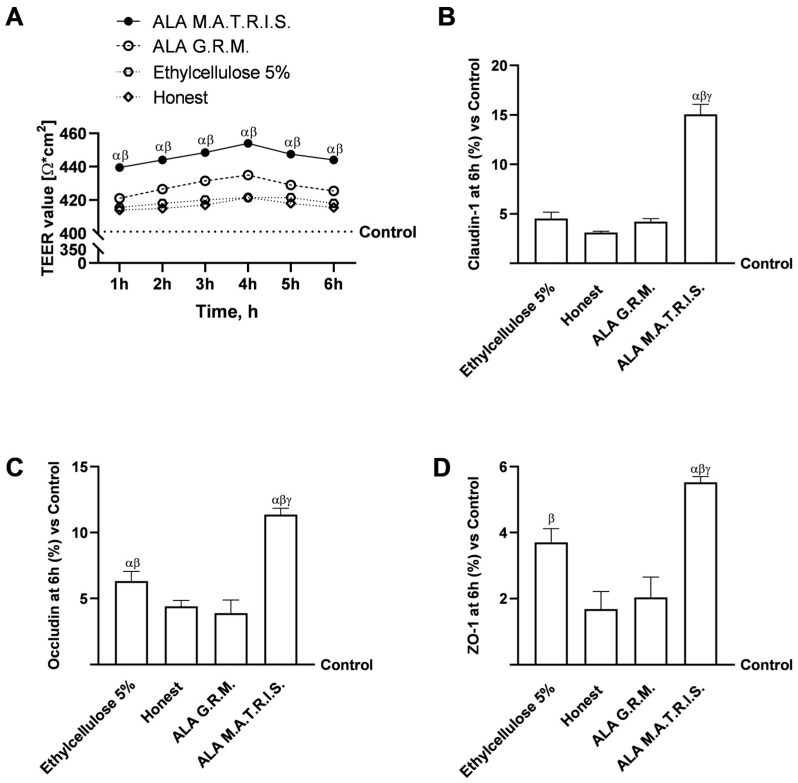
Integrity analysis on in vitro 3D intestinal model after treatment with ALA forms. In (**A**), TEER Value was measured by EVOM3 during the time (from 1 h to 6 h); in (**B**–**D**), the analysis of TJ was measured by ELISA test (Occludin, Claudin-1, and ZO-1, respectively) at 6 h. ALA = α-Lipoic Acid at 50 µM. Data are expressed as mean ± SD (%) of 5 independent experiments, each performed in triplicate, normalised to the control (0%) line. For TEER analysis: *p* < 0.05 vs. Control; α *p* < 0.05 vs. ALA G.R.M.; β *p* < 0.05 vs. excipients (Ethylcellulose 5% and Honest). For TJ analysis: α *p* < 0.05 vs. Control; β *p* < 0.05 vs. ALA G.R.M.; γ *p* < 0.05 vs. excipients (Ethylcellulose 5% and Honest).

**Figure 6:**
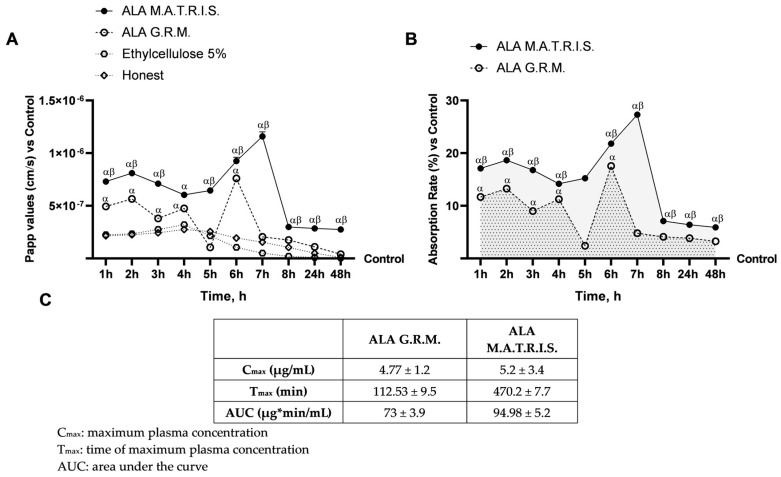
Evaluation of passage through the intestinal barrier after treatment with ALA forms. In (**A**), the apparent permeability coefficient (Papp) values in which data < 0.2 × 10^−6^ cm/s mean very poor absorption with a bioavailability <1%, data between 0.2 × 10^−6^ and 2 × 10^−6^ cm/s with bioavailability between 1 and 90%, and data > 2 × 10^−6^ cm/s mean very good absorption with a bioavailability over 90%; in (**B**), absorption analysis of ALA measured at basolateral level on transwell during time (1 h to 48 h); and in (**C**), pharmacokinetic parameters of the two ALA forms. ALA = α-Lipoic Acid at 50 µM. Data are expressed as mean ± SD (%) of 5 independent experiments, each performed in triplicate, normalised to the control (0%) line. α *p* < 0.05 vs. Control; β *p* < 0.05 vs. ALA G.R.M.

Further experiments were carried out to investigate the absorption kinetics of the two forms of ALA and the maintenance of proper intestinal function and integrity. Therefore, at this stage, a 3D model of the intestinal barrier was set up using the Transwell system to analyse cell viability, radical oxygen species (ROS) production, production of pro-inflammatory cytokines (tumour necrosis factorα, TNFα, and interleukin 1β, IL-1 β), transepithelial electrical resistance (TEER) values and tight junctions (TJs) levels (Figure 4 and Figure 5). In addition, the apparent permeability coefficient, uptake, and ALA plasma concentration-time curve (area under the curve, AUC) after one treatment period were evaluated (Figure 6). As shown in Figure 4A, the viability results obtained by a time-dependent study showed that two forms of ALA were able to increase intestinal cell viability throughout the treatment interval of 1 h to 6 h compared with control (*p* < 0.05) with a peak of activity at 4 h of treatment (17.6% ALA G.R.M. vs. control, *p* < 0.05; 29.7% ALA M.A.T.R.I.S. vs. control, *p* < 0.05) and the excipients (Ethylcellulose 5% and Hones, *p* < 0.05). Although both forms exert a beneficial effect on intestinal cells, the greatest effect was observed after ALA M.A.T.R.I.S. treatment, exceeding the effect exerted by ALA G.R.M. by about 40.5% (*p* < 0.05). In addition, ALA M.A.T.R.I.S. exerted a great effect compared to the excipients alone at 4 h of treatment by about 55% (vs. Ethylcellulose 5%, *p* < 0.05) and by about 51% (vs. Honest, *p* < 0.05). These results can be due to the effects of the M.A.T.R.I.S. technology, which leads to an enhanced delivery and bioavailability of ALA. Results on ROS production also showed a greater beneficial effect from ALA M.A.T.R.I.S. treatment than ALA G.R.M. and excipients (Ethylcellulose 5% and Honest) treatment throughout the treatment interval (Figure 4B), again possibly due to the cooperative effects induced by the use of M.A.T.R.I.S. technology for ALA. Furthermore, to confirm the absence of adverse gut reactions after treatment with the two forms of ALA under study, the inflammatory response at the end of 6 h of treatment was assessed by measuring TNFα and IL-1β production (Figure 4C,D). For both pro-inflammatory cytokines, the two forms of ALA were able to maintain cytokine production below control values (*p* < 0.05) and excipients (Ethylcellulose 5% and Honest, *p* < 0.05); however, of the two forms, the better one was ALA M.A.T.R.I.S as it showed a reduction in TNFα and IL-1β production of about 21% and 16% compared to ALA G.R.M. (*p* < 0.05); even in this case, the more positive data obtained can be ascribed to the combination with M.A.T.R.I.S. technology. In conclusion, these data suggest a kind of cooperation between M.A.T.R.I.S. technology and the raw material ALA.

After these preliminary assessments of gut-level safety, analyses about integrity were conducted, which involved the examination of TEER values alongside evaluating TJ proteins, specifically Claudin-1, Occludin, and ZO-1. As illustrated in Figure 5A, the TEER analysis indicated that both forms of ALA subjected to testing actively facilitated preserved intestinal homeostasis (*p* < 0.05). More precisely, the transit through the intestinal epithelium demonstrated that ALA G.R.M. and ALA M.A.T.R.I.S. upheld epithelial integrity by enhancing the ion flux associated with paracellular transport through the intestinal epithelium relative to the control (*p* < 0.05) and excipients (Ethylcellulose 5% and Honest, *p* < 0.05). The most pronounced effect was documented following the administration of ALA M.A.T.R.I.S. compared to ALA G.R.M. throughout the treatment duration, with peak efficacy observed at the 4-hour mark (*p* < 0.05), in accordance with the hypothesis that there was a cooperative effect between the technology employed and ALA. These findings were corroborated through TJ analysis; as depicted in Figure 5B–D, both ALA M.A.T.R.I.S. and ALA G.R.M. exhibited significantly greater effects than the control and excipients (Ethylcellulose 5% and Honest, *p* < 0.05) across all TJs assessed (*p* < 0.05). Furthermore, ALA M.A.T.R.I.S. demonstrated the most substantial impact when compared to ALA G.R.M. for Claudin-1 (approximately 72%, *p* < 0.05), Occludin (approximately 66%, *p* < 0.05), and ZO-1 (approximately 63%, *p* < 0.05), thanks to the use in combination with M.A.T.R.I.S. technology.

Since the M.A.T.R.I.S. system is a technology based on coating particles of active ingredients with an insoluble and permeable membrane acting by diffusion, the permeability, uptake, and plasma concentration defined as AUC of the two forms of ALA were evaluated. These analyses were necessary to determine how the M.A.T.R.I.S. technology affects and changes the absorption kinetics of ALA compared to the ALA G.R.M. As illustrated in Figure 6A,B, the permeability and adsorption outcomes, assessed within the timeframe of 1 to 48 h, indicated that ALA M.A.T.R.I.S. formulation displayed altered permeability and adsorption kinetics in contrast to ALA G.R.M. formulation. Specifically, whereas the ALA R.G.M. formulation exhibited a peak at 6 h of treatment, the ALA M.A.T.R.I.S. demonstrated a peak at 7 h of treatment. This observed shift in the peak is hypothesized to be attributable to the M.A.T.R.I.S. technology, thereby effectively illustrating the function of this technology as a controlled-release mechanism for active ingredients. To substantiate this assertion, following the execution of the absorption analysis, it became feasible to ascertain the concentration of ALA that successfully traversed the intestinal barrier and reached the basolateral environment. As reported in Figure 6C, the maximum plasma concentration of ALA as a raw material reported in the literature is 7 μg/mL [18], while that obtained from the analyses was 4.77 μg/mL. Similarly, a difference was also observed in the time of ALA uptake; indeed, if ALA absorption is reported in the literature about every 52 min until the maximum plasma concentration is reached [18], ALA G.R.M. tested showed absorption of ALA about every 113 min until maximum plasma concentration was reached (*p* < 0.05). Comparing data in the literature [18] and those obtained, a difference also emerged in AUC determination of about 67 μg/mL between our data and those reported in the literature (the literature: 5.77 ± 0.81 μg ∙ min/mL vs. data obtained: 73 ± 3.9 μg ∙ min/mL, *p* < 0.05). Similarly, a difference was also observed among data reported in the literature for ALA M.A.T.R.I.S. [19]. Indeed, ALA M.A.T.R.I.S. in formulation showed a C_max_, T_max_, and AUC of about 1398 ng/g, 180 min, and 3187 ng × h/g, while tested ALA M.A.T.R.I.S. showed a C_max_, T_max_, and AUC of 5.2 μg/mL, 470 min, and 94.98 μg*min/mL. In conclusion, ALA prepared by M.A.T.R.I.S. technology showed better intestinal activity than ALA G.R.M., confirming how the use of technology is useful for implementing ALA absorption at the intestinal level.

**Figure A2:**
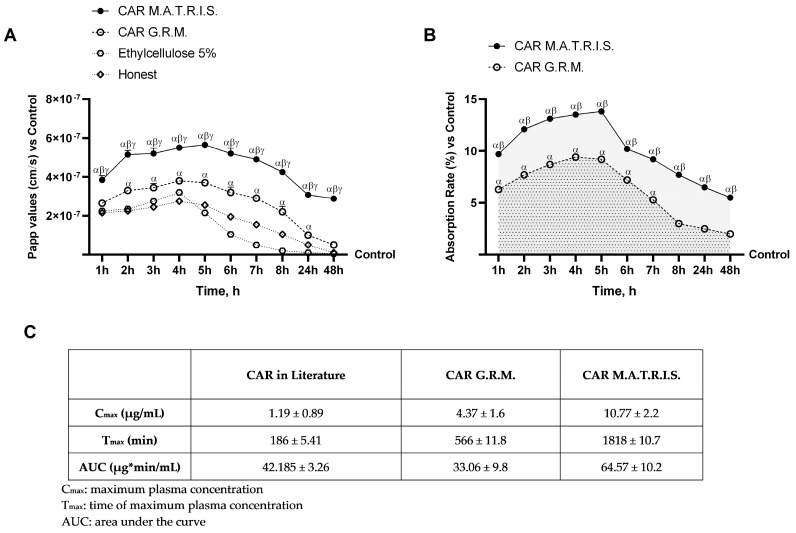
In (**A**), the Papp values, in which data < 0.2 × 10^−6^ cm/s mean very poor absorption with a bioavailability < 1%, data between 0.2 × 10^−6^ and 2 × 10^−6^ cm/s with bioavailability between 1 and 90%, and data > 2 × 10^−6^ cm/s mean very good absorption with a bioavailability over 90%; in (**B**), absorption analysis of CAR measured at basolateral level on transwell during time (1 h to 48 h); and in (**C**), pharmacokinetic parameters of the two CAR forms. CAR = Acetyl-L-Carnitine 7.4 mM. Data are expressed as mean ± SD (%) of 5 independent experiments normalised to the control (0%) line. α *p* < 0.05 vs. Control; β *p* < 0.05 vs. CAR G.R.M.; γ *p* < 0.05 vs. excipients (Ethylcellulose 5% and Honest).

To give value to this technology, the same analyses of permeability, absorption, and determination of AUC were also performed on the two forms of CAR (Figure A2 reported in Appendix A). As illustrated in Figure A2A,B, the permeability and adsorption outcomes, assessed within the timeframe of 1 to 48h, indicated that CAR M.A.T.R.I.S. formulation displayed altered permeability and adsorption kinetics in contrast to CAR G.R.M. specifically. In contrast, the CAR G.R.M. formulation exhibited a peak at 6 h of treatment; the CAR M.A.T.R.I.S. formulation demonstrated a peak at 5 h of treatment. This observed shift in the peak is hypothesised to be attributable to the M.A.T.R.I.S. technology, thereby effectively illustrating the function of this technology as a controlled-release mechanism for active ingredients. To substantiate this assertion, following the execution of the absorption analysis, it became feasible to ascertain the concentration of CAR that successfully traversed the intestinal barrier and reached the basolateral environment. As reported in Figure A2C, the maximum plasma concentration of CAR as a raw material reported in the literature is 1.19 μg/mL [20], while that obtained from the analyses was 4.37 μg/mL. Similarly, a difference was also observed in the time of CAR uptake; indeed, if CAR absorption is reported in the literature about every 186 min until the maximum plasma concentration is reached [20], CAR G.R.M. tested showed absorption of CAR about every 566 min until maximum plasma concentration was reached (*p* < 0.05). Comparing data in the literature [20] and those obtained, a difference also emerged in AUC determination of about 33.06 μg/mL between our data and those reported in the literature (the literature: 42.185 ± 3.26 μg*min/mL vs. data obtained: 33.06 ± 9.8 μg*min/mL, *p* < 0.05). Regarding CAR M.A.T.R.I.S. in formulation, it showed a C_max_, T_max_, and AUC of 10.77 μg/mL, 1818 min, and 64.57 μg*min/mL. In conclusion, CAR prepared by M.A.T.R.I.S. technology showed better intestinal activity than CAR G.R.M., confirming how the use of technology is useful for modulating CAR absorption at the intestinal level.

### 2.3. Effects of ALA G.R.M. And ALA M.A.T.R.I.S. on 3D EngNT Model

**Figure 7:**
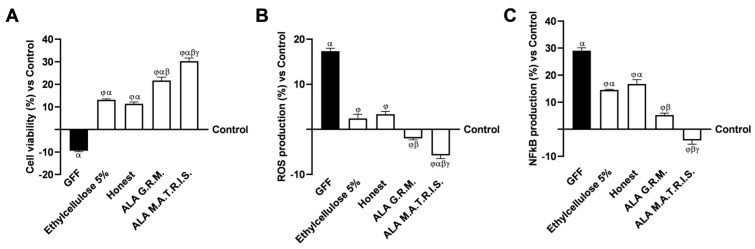
Effects of ALA G.R.M. and ALA MATRIS on 3D EngNT model. In (**A**), cell viability was measured by MTT test at 24 h; in (**B**), the analysis of ROS production was measured by cytochrome C reduction at 24 h; in (**C**), NFkB production was measured by specific ELISA Kit. ALA = α-Lipoic Acid at 50 µM. Data are expressed as mean ± SD (%) of 5 independent experiments, each performed in triplicate, normalised to the control (0%) line. α *p* < 0.05 vs. Control; β *p* < 0.05 vs. ALA G.R.M.; γ *p* < 0.05 vs. excipients (Ethylcellulose 5% and Honest); φ vs. GGF 200 ng/mL.

To reproduce peripheral nerve tissue injury in vitro, EngNT 3D was exposed to a pretreatment regimen incorporating 200 ng/mL of Glial Growth Factor (GGF), initiated on day 14 of the maturation timeline, to enable extensive demyelination before administering the aforementioned agents. Furthermore, supplementary assessments were conducted to investigate the effects on cell viability and ROS production within this context, as delineated in Figure 7A,B. Nerve tissue treated with 200 ng/mL GGF showed a substantial decrease in nerve biological activity and increased ROS production compared to the control (*p* < 0.05). On the other hand, the negative conditions were restored after treatment with the two forms of ALA (*p* < 0.05). In particular, ALA M.A.T.R.I.S. was able to statistically significantly improve cell viability compared with the ALA G.R.M. form (about 28%, *p* < 0.05) and the excipients (about 57% Ethylcellulose 5% and 62% Honest, *p* < 0.05). In addition, ALA M.A.T.R.I.S. could also reduce oxidative stress (about 65.5%, *p* < 0.05) produced during damage conditions, supporting the previously observed results. The better results obtained with ALA M.A.T.R.I.S can be ascribable to the use of ALA in combination with the novel technology. To accomplish analyses at this level, the inflammatory response activated following damage induction and the reducing action of the two forms of ALA on this activation were evaluated. Nuclear factor kappa–light–chain enhancer of activated B cells (NFkB) production (Figure 7C) exhibited a significant increase following damage induction with GGF 200 ng/mL in comparison to the control group (*p* < 0.05). Conversely, treatment with ALA G.R.M. and ALA M.A.T.R.I.S. led to a substantially reduced NFkB production compared to damage induced by GGF 200 ng/mL (*p* < 0.05). Specifically, the greatest beneficial effect was observed after treatment with ALA M.A.T.R.I.S. compared with the form without technology and excipients (about 2.28-fold, *p* < 0.05; about 4.53-fold Ethylcellulose 5% and 5.07-fold Honest, *p* < 0.05), confirming the possible amplification of the power of restoration of the damage condition due to the improvement in ALA absorption related to M.A.T.R.I.S. technology.

**Figure 8:**
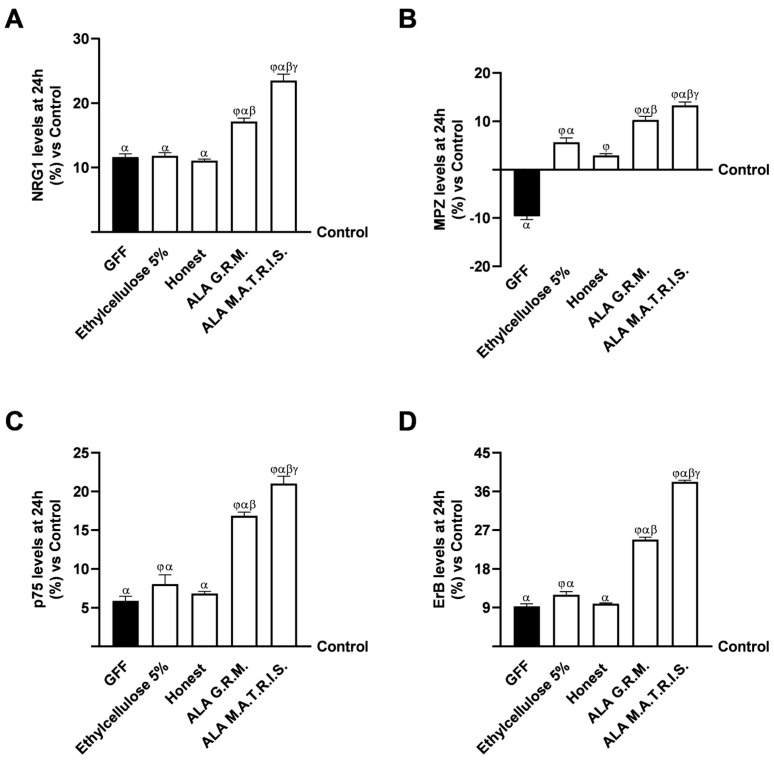
Effects of ALA G.R.M. and ALA M.A.T.R.I.S. on 3D EngNT model. In (**A**), NRG1 levels were measured by a specific ELISA Kit; in (**B**), MPZ levels were measured by a specific ELISA Kit; in (**C**), p75 levels were measured by a specific ELISA Kit; and in (**D**), ErB levels were measured by specific ELISA Kit. ALA = α-Lipoic Acid at 50 µM. Data are expressed as mean ± SD (%) of 5 independent experiments, each performed in triplicate, normalised to the control (0%) line. α *p* < 0.05 vs. Control; β *p* < 0.05 vs. ALA G.R.M.; γ *p* < 0.05 vs. excipients (Ethylcellulose 5% and Honest); φ vs. GGF 200 ng/mL.

Finally, the mechanisms regulating specific molecular pathways related to restoring peripheral well-being after treatment with 200 ng/mL of GGF were analysed (Figure 8). As shown in Figure 8A, after treatment with the two forms of ALA, an improvement in nerve injury is observed. Specifically, treatment with ALA M.A.T.R.I.S. showed a significant beneficial effect on neuregulin 1 (NRG1) levels compared to untreated cells (about 23.5% compared to the control, *p* < 0.05) to those placed in damaged condition and those treated with the form of ALA G.R.M. (about 27%, *p* < 0.05) and excipients (about 49% Ethylcellulose 5% and 53% Honest, *p* < 0.05). This profile has also been observed on protein levels maintaining the myelinic sheath (myelin protein-zero, MPZ, Figure 8B). Both forms of ALA improved MPZ levels compared to GGF (*p* < 0.05), indicating the restoration of myelin sheath; more precisely, ALA M.A.T.R.I.S. induced greater effects than the form ALA G.R.M. (about 23%, *p* < 0.05) and excipients (about 57.6% Ethylcellulose 5% and 78% Honest, *p* < 0.05), reversing the induced damage. Similar to what has been observed so far, the analysis of p75 expression confirmed these data (Figure 8C). After pretreatment with GGF, the myelinating cells were degraded, but this condition was restored after treatment with the two forms of ALA (*p* < 0.05). In particular, ALA M.A.T.R.I.S. maintained the normal activity of the myelin sheath, increasing the expression of p75 better than ALA G.R.M. (about 20%, *p* < 0.05) and excipients (about 62% Ethylcellulose 5% and 67.5% Honest, *p* < 0.05), correcting the damage and inducing a restoration of activity even compared to control (about 21%, *p* < 0.05). In conclusion, the treatment with the two forms of ALA demonstrated a significant impact on the level of the beta receptor (ERb), as evidenced by its ability to reduce the damage induced by GGF (Figure 8D). This finding serves to reinforce the notion that these forms of ALA possess a beneficial role in the prevention of demyelination (*p* < 0.05). Specifically, the level of ERb increased in the presence of ALA M.A.T.R.I.S. compared to ALA G.R.M. (about 35%, *p* < 0.05) and excipients (about 69% Ethylcellulose 5% and 74% Honest, *p* < 0.05), thereby supporting the hypothesis that better control intestinal release due to the technology may enhance the effects of drugs on the specific target such as on specific markers involved in the restoration of nerve injury and the process of myelination. In conclusion, it can be posited that controlled intestinal release can influence several intracellular mechanisms related to the ability of the specific substances treated with M.A.T.R.I.S. to reduce the negative condition associated with the substances.

**Figure A3:**
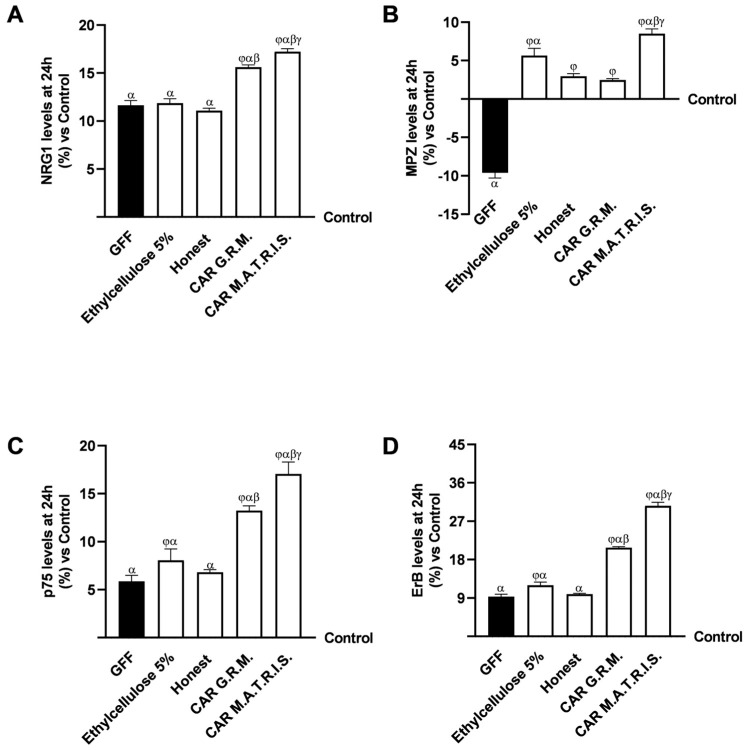
Effects of CAR G.R.M. and CAR M.A.T.R.I.S. on 3D EngNT model. In (**A**), NRG1 levels were measured by a specific ELISA Kit; in (**B**), MPZ levels were measured by an ELISA Kit; in (**C**), p75 levels were measured by an ELISA Kit; and in (**D**), ErB levels measured by specific ELISA Kit. CAR = Acetyl-L-Carnitine 7.4 mM. Data are expressed as mean ± SD (%) of 5 independent experiments normalised to the control (0%) line. α *p* < 0.05 vs. Control; β *p* < 0.05 vs. CAR G.R.M.; γ *p* < 0.05 vs. excipients (Ethylcellulose 5% and Honest); φ vs. GGF 200 ng/mL.

Similar to what was observed for the ALA forms, the two forms of CAR tested at the peripheral level also showed interesting data (Figure A3 in Appendix A). Specifically, the two forms of CAR after intestinal transit were able to modulate the mechanisms involved in the maintenance of peripheral well-being, with a greater beneficial effect being observed after treatment with CAR M.A.T.R.I.S. compared with CAR G.R.M. (NRG1: about 10%, *p* < 0.05; MPZ: 71%, *p* < 0.05; p75: 22%; and Erb: 32%, *p* < 0.05).

In conclusion, based on all the data obtained, it is possible to hypothesise a synergistic effect between ALA and M.A.T.R.I.S. technology, as the latter appears to significantly enhance the biological effects of ALA. This enhancement can be attributed to the distinctive features of M.A.T.R.I.S. technology, which modulates the release kinetics of the molecule, resulting in a more sustained and targeted delivery.

## 3. Discussion

As a food supplement with positive effects in treating several conditions, ALA and CAR have drawn much attention throughout time [21]. ALA has clinically valuable properties, including acting as an enzymatic cofactor [22], being involved in glucose and lipid metabolism, managing gene transcription, acting as an antioxidant, and efficiently removing heavy metals from the bloodstream [9]. On the other hand, the therapeutic potential of CAR for neuroprotection in a variety of conditions, such as traumatic brain injury, Alzheimer’s disease, and disorders causing damage to the central or peripheral nervous systems, has attracted a lot of attention in recent years [23]. Both ALA and CAR are available in a variety of oral formulations, with ALA available in film-coated tablets, soft capsules, hard capsules, powder, and aqueous solutions, in the worldwide dietary supplement market due to its well-established positive effects [24], and CAR is used worldwide for various nutritional and pharmaceutical applications due to its highly therapeutically efficacy [25]. Despite having a variety of biological activities, ALA’s pharmacokinetic profile has led to limited therapeutic efficacy in several studies. Indeed, data point to a short half-life and bioavailability of roughly 30% because of several mechanisms, such as hepatic degradation, decreased solubility, and stomach instability [26]. However, this can be further enhanced by using a variety of novel formulations that directly raise ALA’s bioavailability. After oral administration, ALA was shown to be quickly absorbed, and it took roughly 30 min to 1 h to reach the highest plasma concentrations [27]. Regarding CAR, following oral administration, it is absorbed partially through passive diffusion and partially through carrier-mediated transport, but 5–18% is the absolute bioavailability following oral dosages of 1–6 g. Because of the slow mucosal uptake and extended mucosal retention that characterise CAR absorption, it takes 4 to 6 h for individuals to reach their maximal plasma concentrations following oral administration [28].

In this context, the development of M.A.T.R.I.S. technology is capable of increasing absorption and bioavailability but mainly promoting an important controlled release over time. Indeed, M.A.T.R.I.S. represents the state of the art in the field of oral sustained-release active ingredients. It features a homogeneous and maximum dispersion of the active ingredient over the entire area of the gastrointestinal tract, ensuring uniform absorption during the established period. Therefore, this study aimed to analyse the effect of the new M.A.T.R.I.S. technology applied to ALA and CAR to evaluate its effect at the neuropathic level following intestinal metabolism. Evaluation of this new technology has shown that the chemical and morphological characterisation of ALA and CAR before and after the granules have been coated with M.A.T.R.I.S. technology remains similar, as evident from SEM images. X-ray diffraction and FT-IR/ATR spectroscopy analyses also show that the profile of the ALA G.R.M. is comparable to that of ALA M.A.T.R.I.S. Following the identification of similarity between the two formulas, ALA G.R.M. and ALA M.A.T.R.I.S. were studied at the intestinal level. At the intestinal level, not only does ALA M.A.T.R.I.S. maintain the integrity of the intestinal barrier more effectively, but what is more important is that the use of this technology modulates the absorption and release of ALA over time compared to ALA G.R.M. Several studies have shown that considering the subject’s absorption, the bioavailability of ALA at the gastrointestinal level appears to be subject to variability. Indeed, administration of ALA concomitantly with food results in a reduction in its bioavailability, a decline in the maximum plasma concentration (C_max_) of approximately 30%, and a decrease in the total concentration of ALA by approximately 20% in comparison with its intake on an empty stomach [9]. Additionally, the pharmacokinetic limitations of ALA following oral administration have been demonstrated to reduce its therapeutic efficacy. These limitations include reduced solubility, lack of gastric stability, and hepatic degradation, resulting in a bioavailability of approximately 30% and a short half-life for ALA. A pharmacokinetic profile analysis of a liquid formulation of ALA revealed that it is absorbed more rapidly with higher plasma concentrations and demonstrates greater stability after gastric transit compared to a solid formulation [26]. However, very different data were obtained with the M.A.T.R.I.S. technology; indeed, almost 30% of ALA M.A.T.R.I.S. is absorbed after 7 h of treatment, thus greatly extending the timing of ALA absorption using this technology. Data on the AUC, or the level of drug exposure, also correlates with the absorption data: this can be seen by the difference in AUC value between ALA G.R.M. and ALA M.A.T.R.I.S. These findings demonstrate the value of using M.A.T.R.I.S. technology to implement ALA action at the intestinal level. Similar data were obtained when CAR was analysed. Indeed, the M.A.T.R.I.S. technology induced a change in the absorption peak between the two formulations, confirming the ability of this technology to act as a controlled release mechanism for active ingredients. Even the AUC analysis has confirmed a change in the mechanism after utilising the M.A.T.R.I.S. technology.

Finally, the last target of this study was nerve tissue in damaged conditions. ALA, being a natural compound rich in antioxidants, has been shown to effectively treat neuropathic pain due to its ability to control neuropathic pain-related symptoms [27,29]. By preventing oxidative damage, antioxidant molecules such as ALA work on a variety of targets and pathways, ultimately helping to repair nerve dysfunction [30]. Also, CAR has demonstrated some ability to counteract neuropathic pain of different etiologies [31]. Indeed, some studies have confirmed that CAR administration can improve pain perception in people affected by peripheral neuropathy [32]. Therefore, the ability of ALA and CAR to act at the neuropathic level is already recognised in the literature. This was demonstrated by the fact that ALA Granular Raw Material reversed the production of ROS and inflammatory cytokines compared to the damaged state. On the other hand, as additional evidence of ALA M.A.T.R.I.S.’s superior effectiveness, the latter substantially enhanced the outcomes of ALA G.R.M. in every parameter examined (*p* < 0.05). Because it is involved in many different Schwann cell functions, the neuregulin1 (NRG1)/ErbB signalling pathway is thought to control Schwann cell plasticity [33]. Important functions such as Schwann cell survival, proliferation, migration, differentiation, myelination, and demyelination are regulated by the NRG1/ErbB pathway [33,34]. The treatment with both forms of ALA restored the altered neurotropism induced by neuropathy condition, also preventing damage to motor fibres and slowing down nerve conduction, demonstrating the neurotropic properties of ALA. Once more, the higher efficacy of ALA made using M.A.T.R.I.S. technology can be supported because, in comparison to ALA G.R.M., this preparation was able to dramatically raise NRG1 and ErbB levels (*p* < 0.05). Numerous pharmacological investigations indicate that by upregulating MPZ expression, nerve injury can be repaired and neuron regeneration enhanced [35,36]. Since it has been demonstrated to restore MPZ levels in the damage model, this mechanism of action can also be linked to ALA. Once more, more noticeable effects have been rendered possible by utilising innovative M.A.T.R.I.S. technology (*p* < 0.05). Correlated to the increase in MPZ protein following the treatment with the two ALA forms, there is an increase in p75 levels, demonstrating modulation of specific molecular pathways involved in repristinating the myelinisation following nerve injury. Once more, the findings on the effectiveness of M.A.T.R.I.S. technology were shown using CAR in a peripheral system study, as CAR M.A.T.R.I.S. was able to increase the expression of NRG1, p75, ErbB, and MPZ, indicating that nerve damage conditions could be improved.

M.A.T.R.I.S. technology seems to improve the already known beneficial activity of ALA and CAR, particularly its antioxidant mechanisms, including modulating several signalling pathways. These results suggest that the new M.A.T.R.I.S. technology may enhance the substances’ ability to cross biological membranes, particularly the intestinal barrier, and reach their intended site, as observed by the best results in the in vitro models. Indeed, the M.A.T.R.I.S. technology is intended to enhance the cellular uptake and prolong the bioavailability of ALA at the site of action, thereby amplifying its biological impact in comparison with the untreated molecule. This synergy can be attributed to the structural and physicochemical characteristics of the delivery system, which may facilitate more effective intracellular penetration, retention and signalling. Nonetheless, it is imperative to acknowledge that these assumptions must be substantiated and validated in future research.

Despite the considerable potential and promise demonstrated by the technology under investigation in this study for future applications, it is imperative to acknowledge that it remains at an early stage of investigation. This is primarily due to the lack of comparative data on other technologies that have already been released, are in active use, and have been rigorously tested and validated in the field. Moreover, it is important to acknowledge the preliminary nature of the analysis and exploration conducted in this study, as well as the lack of validation of the data in an in vivo model.

## 4. Materials and Methods

### 4.1. Agents Preparation

The cells were treated with 50 μM [37] of two different ALA forms and 7.4 mM of two different CAR forms (donated by I.P.S. srl, San Giuliano Milanese, Italy) dissolved in Dulbecco’s Modified Eagle’s Medium without red phenol (DMEM; GIBCO^®^ ThermoFisher Scientific, Waltham, MA, USA) and supplemented with 2 mM L-glutamine and 1% penicillin–streptomycin. The first ALA and CAR were the starting raw materials (named G.R.M.). In contrast, the second form of ALA and CAR was prepared with M.A.T.R.I.S. technology (produced by I.P.S. srl, San Giuliano Milanese, Italy) [8]. Briefly, the active substance coating process involves placing 3.0 kg of starting material in a 10 L rotating pan. While the pan is rotating, the active substance is immersed in a solution consisting of 300 g of shellac 30% in ethanol and 300 g of talcum powder. The coating operation can be carried out continuously or in several stages until the desired release rate is achieved. Next, the product undergoes a sieving process with a 790 μm mesh, followed by a dusting process with a 425 μm mesh. At the end of this process, the product is left to dry in the tank for three hours at room temperature. The M.A.T.R.I.S. technology has been patented in Europe and the USA to allow active substances to spread quickly and evenly over the entire surface of the gastrointestinal tract by initiating modified release at the intestinal and peripheral nervous levels. In addition, the excipients Ethylcellulose 5% and Honest used to create the M.A.T.R.I.S. form were tested alone to study their influence on absorption. Also, the Glial Growth Factor (GGF, Tebu-Bio, Magenta, Milan, Italy) was added directly to the medium in the 3D EngNT to induce demyelination at the final concentration of 200 ng/mL.

### 4.2. SEM Analysis

The ALA and CAR samples were observed using an FEI electron microscope (Field Electron and Ion Company, Hillsboro, OR, USA) coupled with elemental analysis (EDX) with an electron beam accelerated at 25 kV [38,39,40].

### 4.3. X-Ray Diffraction

The crystalline profile of the ALA samples was acquired using a “Miniflex 600” diffractometer from Rigaku (Matsubara, Tokyo, Japan). The analysis was recorded with the following instrumental parameters:

- 2θ range: 3–40 degrees;
- Step: 0.02 degrees 2θ;
- Scanning time: 1 s;
- Intensity, voltage: 15 mA, 40 kV.

### 4.4. Thermal Analysis—Differential Scanning Calorimetry

The ALA samples were subjected to programmed heating in a nitrogen flow from 25 °C to 250 °C (10 °C/min). A “DSC3” apparatus from Mettler Toledo (Greifensee, CH) was used.

### 4.5. FT-IR/ATR Spectroscopy

IR spectra were recorded with Perkin Elmer “Spectrum Two” equipment (Schelton, Connecticon, USA) in the range 4500–400 cm^−1^ with a resolution of 4 cm^−1^ on ALA samples.

### 4.6. Cell Culture

The human epithelial intestinal, Caco-2, cell line was used as a model to predict the features of human intestinal absorption, and it is widely used to study the mechanism of drugs after oral intake in humans [41,42]. Cells were supplied by American Type Culture Collection (ATCC) and cultured in Advanced Dulbecco’s Modified Eagle’s Medium/Nutrient F-12 Ham (Adv DMEM-F12; GIBCO^®^ ThermoFisher Scientific, Waltham, MA, USA) supplemented with 10% FBS, 2 mM L-glutamine, and 1% penicillin–streptomycin, maintained in an incubator at 37 °C and 5% CO_2_ and 95% of humidity [43]. Experiments were performed using Caco-2 cells at passage numbers between 26 and 32 to maintain the physiological balance among paracellular permeability and transport properties [44]. Cells were plated in different ways, based on different experimental protocols: 1 × 10^4^ cells on 96 well plates to study cell viability by MTT-based In Vitro Toxicology Assay Kit (Merck Life Science, Rome, Italy) and ROS production synchronising cells for 8 h with DMEM without red phenol and supplemented with 0.5% FBS, 2 mM L-glutamine, and 1% penicillin–streptomycin at 37 °C; 2 × 10^4^ cells on 6.5 mm Transwell^®^ (Corning^®^ Costar^®^, Merck Life Science, Rome, Italy) with a 0.4 μm pore polycarbonate membrane insert (Corning^®^ Costar^®^, Merck Life Science, Rome, Italy) in a 24-well plate to perform the apparent permeability coefficient (Papp) analysis, integrity analyses by transepithelial electrical resistance (TEER) and tight junction (TJ) evaluation, absorption analysis, and area under the curve (AUC) determination.

Rat-derived Schwann, RSC-96 cell line (purchased from ATCC) was cultured in Adv DMEM (GIBCO^®^ ThermoFisher Scientific, Waltham, MA, USA) supplemented with 10% FBS, 2 mM L-glutamine, and 1% penicillin–streptomycin [45] maintained in an incubator at 37 °C, 5% CO_2,_ and 95% humidity. Experiments were performed using RSC96 cells at passages between 10 and 15, subcultured 2–3 times a week.

The rat neuronal PC12 cell line, supplied by ATCC, was cultured in Roswell Park Memorial Institute-1640 (RPMI, Merck Life Science, Rome, Italy) supplemented with 2 mM glutamine, 10% horse serum (HS; Merck Life Science, Rome, Italy), and 5% FBS. The cultures were maintained at sub-confluency in an incubator at 37 °C with 5% CO_2_ and 95% humidity. The cells used for the experiments had been passaged between 3 and 13 times [46]. The conditions with 4 × 10^6^ RSC96 cells and 1 × 10^5^ PC12 cells were the most appropriate to seed a co-culture system to reproduce in vitro the 3D EngNT in the peripheral nerve environment [45].

### 4.7. Experimental Protocol

The research project was divided into two phases. The initial phase investigated the impacts of two distinct forms of ALA and CAR on a 3D intestinal model, which is created by seeding Caco-2 intestinal cells on a Transwell^®^ support for 21 days, facilitating the establishment and maturation of the cellular monolayer, accompanied by the subsequent formation of intestinal villi. In this 3D intestinal barrier model, cell viability (only for ALA samples), ROS production (only for ALA samples), absorption, and permeability were analysed (for ALA and CAR samples), and the TEER values were measured (only for ALA samples) in a time-dependent study. In addition, the maintenance of a correct TJ activity was evaluated to verify the integrity of the cell monolayer. For all experiments, the intestinal cells were treated for 1 to 6 h with two different forms of ALA and CAR [43]. This treatment interval between 1 and 6 h was chosen to mimic the time of filling and emptying of the intestine. Still, the treatment has been extended to 48 h for permeability, absorption analysis, and AUC determination in order to assess the technology’s modulation of absorption.

Moreover, at the end of each stimulation, the basolateral environment was collected to stimulate the 3D EngNT co-culture. Indeed, in the last step, the 3D EngNT co-culture was used to study the effects of stimulations on the in vitro nervous tissue model after 14 days of maturation of the culture in a condition of demyelination, induced by 200 ng/mL GGF added to the 3D EngNT co-culture from day 14; this step is a well-established model for mimicking peripheral neuronal injury [47]. At this level, cell viability (only for ALA samples), ROS production (only for ALA samples), inflammatory markers (only for ALA samples), re-myelination mechanisms and peripheral nerve recovery mechanisms such as p75, NRG1, MPZ and ERb were analysed (for ALA and CAR samples).

Both cell types were synchronised for 8 h with DMEM without red phenol and supplemented with 0.5% FBS, 2 mM L-glutamine, and 1% penicillin–streptomycin maintained in an incubator at 37 °C with 5% CO_2_.

It is important to note that the comprehensive data acquired from the area under the curve (AUC) assessments and the significant peripherally activated markers associated with the two distinct forms of CAR have been reported in Appendix A for further examination and scrutiny by interested researchers and practitioners in the field.

### 4.8. Cell Viability

Cell viability based on the In Vitro Toxicology Assay Kit (Merck Life Science, Rome, Italy) was assessed at the end of each stimulation with ALA samples, following a classical protocol reported in the literature [48]. The absorbance of each solubilised sample was evaluated at 570 nm with correction at 650 nm, measured by a spectrometer (Infinite 200 Pro MPlex, Tecan, Männedorf, Switzerland). The results were expressed by comparing them to the control sample (untreated samples defined as the 0% line) and reported as the means of five independent experiments performed in triplicate.

### 4.9. ROS Production and Measurement

Superoxide anion release was quantified after treatment with ALA samples using a standard methodology based on cytochrome C reduction [49]. A Tecan spectrophotometer was used to detect the absorbance in culture supernatants at 550 nm after adding 100 μL of cytochrome C (Merck, Milan, Italy) to each well. Comparatively, empty wells were filled with 100 μL of superoxide dismutase (Merck, Milan, Italy) and 100 μL of cytochrome C, and the plate was incubated for 30 min. The O2 rate was quantified as the average standard deviation (%) of nanomoles per decreased cytochrome C per microgram of protein relative to the control (0 line).

### 4.10. Intestinal Barrier In Vitro Model

To assess whether ALA and CAR in different forms could cross the intestinal barrier, an in vitro barrier was created using the Transwell^®^ system following a standard protocol reported in the literature [50,51]. The FDA and the European Medicines Agency (EMA) approved this model to predict the absorption, metabolism, and bioavailability of various substances after oral intake in humans.

Briefly, Caco-2 cells were plated as previously described and kept in a complete medium for 21 days before the simulations, with changes made every other day on the basolateral and apical sides [51]. Throughout the development period, the TEER values were assessed using EVOM3 in conjunction with STX2 chopstick electrodes (World Precision Instruments, Sarasota, FL, USA) to assess the creation of mature intestinal epithelium and an appropriate paracellular mechanism. On the 21st day, when TEER values were 400 Ω*cm^2^ [52], absorption analysis commenced. Before the stimulation, on the apical side, the medium was brought to pH 6.5, the pH in the lumen of the small intestine, while pH 7.4 on the basolateral side represented blood [52]. The cells were stimulated with all substances from 1 to 48 h for the intestinal model before the successive analyses, including the permeability assay measured by Papp (cm/s) analysis [53], which follows the following formula:

Papp = dQ/dt ⇥ 1/m0 ⇥ 1/A ⇥ V Donor

dQ: amount of substance transported (nmol or μg);

dt: incubation time (s);

m0: amount of substrate applied to the donor compartment (nmol or μg);

A: surface area of Transwell^®^ membrane (cm^2^);

V Donor: volume of the donor compartment (cm^3^).

Negative controls without cells were tested to exclude the Transwell^®^ membrane’s influence. The analysis was performed in triplicate and reproduced five times.

### 4.11. TJs Analysis

After treatment with ALA samples, the human occludin (OCLN) ELISA kit (MyBiosource, San Diego, CA, USA), claudin-1 (ELISA kit, Cusabio Technology LCC, Houston, TX, USA), and ZO-1 (human tight junction protein 1 (TJP1) ELISA kit (MyBiosource, San Diego, CA, USA) were used to analyse the CaCo-2 lysates following the manufacturer instructions. The spectrophotometer used to measure absorbance was the Infinite 200 Pro MPlex from Tecan, located in Männedorf, Switzerland, operating at a wavelength of 450 nm. The data were acquired by comparing the standard curve ranging from 0 to 1500 pg/mL for occludin and from 0 to 1000 pg/mL for claudin-1 and ZO-1. The data were shown as a percentage compared to the control (0 lines) from five independent experiments conducted in triplicate [52].

### 4.12. TNF-α ELISA Assay

After treatment with ALA samples, TNFα quantification was obtained using the TNF-α ELISA kit (Merck Life Science, Milano, Italy) according to the manufacturer’s instructions [54]. The absorbance of the samples was measured at 450 nm using a plate reader (Infinite 200 Pro MPlex, Tecan, Männedorf, Switzerland), and the results were expressed as a mean ± SD (%) versus the control (0 line) of five independent experiments performed in triplicate.

### 4.13. Interleukin-1β ELISA Assay

After treatment with ALA samples, the level of IL-1β was quantified using an IL-1β ELISA kit (FineTest, Wuhan, China) according to the manufacturer’s instructions. Briefly, the plate was incubated at 37 °C for 90 min after 100 µL of each sample was added to each well. Following the incubation period, each well’s contents were removed, and Wash Buffer was used twice to wash the wells. The wells were filled with 100 µL of biotin-labelled antibody working solution, and the plate was incubated for 60 min at 37 °C. Following the incubation period, the solution in each well was withdrawn, and Wash Buffer was used three times to wash the wells. After adding 100 µL of SABC Working Solution to each well, the plate was incubated for 30 min at 37 °C. Following five rounds of washing, 90 µL of TMB substrate was added to each well. A plate reader (Infinite 200 Pro MPlex, Tecan, Männedorf, Switzerland) was used to read the plate at 450 nm as soon as 10–20 min had passed after adding 50 µL of Stop Solution to each well. Five independent experiments were conducted in triplicate, and the findings were expressed as mean ± SD (%) versus control (0 line) after the data were gathered and compared to the standard curve (range from 31.25–2000 pg/mL).

### 4.14. Area Under the Curve (AUC) Evaluation

The ALA and CAR samples were taken at the following times: 1, 2, 4, 5, 6, 7, 8, 24, and 48 h post-stimulation. The following pharmacokinetic parameters of ALA and CAR were calculated: the maximum plasma concentration (C_max_); time of maximum plasma concentration (T_max_); terminal half-life (t½:); area under the plasma concentration-time Curve from time zero up to time t (AUC_t_), where t is the last time point at which the subject showed concentrations above the lower limit of quantification, i.e., time of last measurable (non-zero) concentration (t-last) using the trapezoidal rule; area under the plasma concentration–time curve from time of dosing extrapolated to infinity using the trapezoidal rule (AUC_inf_). AUCs were computed using the Log Linear Method, trapezoidal when Cn > Cn^−1^. This study was carried out under quality assurance and quality control systems with written Standard Operating Procedures (SOP) by the Good Clinical Practice (GCP) Guidelines (CPMP/ICH/135/95). Quality assurance was guaranteed by regular monitoring of this study by a qualified monitor [18].

### 4.15. D EngNT In Vitro Model

The 3D nerve tissue model was developed based on the literature [52]. Interactions between RSC96 and PC12 cell lines are crucial for replicating the peripheral nerve environment in vitro, promoting neurite regeneration, and supporting Schwann cells. In summary, a rectangular scaffold measuring 16.4 mm × 6.5 mm × 5 mm was filled with 1 mL of a solution that contained 80% *v*/*v* Type I rat tail collagen (2 mg/mL in 0.6% acetic acid, Thermo Fischer, Milan, Italy), 10% *v*/*v* Minimum Essential Medium (MEM, Merck Life Science, Milano, Italy), 5.8% *v*/*v* neutralising solution (Biosystems, Monza, Italy), and 4.2% Schwann cell suspension (4 × 10^6^ RSC96 cells per 1 mL gel). After the gel solidified, it was submerged in 10 mL of DMEM (Merck Life Science, Rome, Italy) with red phenol and supplemented with 10% FBS, 100 U/mL of Penicillin, and 100 μg/mL of Streptomycin (Merck Life Science, Milano, Italy) for 24 h at 37 °C with 5% CO_2_. Upon completion of the incubation period, the gel was stabilised using plastic compression (120 g of weight per minute). The gel was divided into equal parts based on the samples to be treated once it had been aligned and stabilised. After the aligned Schwann gels, each gel segment was moved to a 24-well plate. To construct the co-cultures, 1 × 10^5^ PC12 was seeded on top of each segment. This passage is crucial since it enables neurite outgrowth horizontally. After allowing neuronal cells to attach to the collagen gel by incubating the 24-well plate with gels for one hour at 37 °C, 1 mL of culture medium was added to each well.

### 4.16. NFkB ELISA Assay

After treatment with ALA samples, the NF-κB Transcription Factor Assay Kit was used to measure the level of NFkB (Cayman Chemical Company, Ann Arbor, MI, USA), following the manufacturer’s instructions [55]. Briefly, nuclear extracts were prepared using a nuclear extraction protocol, and NFKB contained in these extracts was detected by the addition of a specific primary antibody. A secondary antibody conjugated to HRP is added to provide a sensitive colourimetric measurement by a spectrometer (Infinite 200 Pro MPlex, Tecan, Männedorf, Switzerland) at 450 nm. Five independent experiments were conducted in triplicate, and the findings were expressed as mean ± SD (%) versus control (0 line) after the data were gathered and compared to the standard curve.

### 4.17. p75 ELISA Assay

After treatment with ALA and CAR samples, the Rat NGFR ELISA kit (MyBiosource, San Diego, CA, USA) was utilised on 3D EngNT lysates following the manufacturer’s instructions [56]. Using a spectrophotometer (Infinite 200 ProMPlex, Tecan, Männedorf, Switzerland), the plate was read at 450 nm. The acquired data were compared to the standard curve, which ranges from 0.312 to 20 ng/mL. The findings were presented as the mean ± SD (%) of five separate, triplicate tests compared to the control (0 lines).

### 4.18. MPZ ELISA Assay

After treatment with ALA and CAR samples, using a Rat ELISA kit (MyBiosource, San Diego, CA, USA) for cell lysates, the synthesis of myelin protein zero (MPZ) was measured following the manufacturer’s instructions [57]. In brief, 100 µL of material was added to each well, and the plate was incubated at 37 °C for two hours. Following the conclusion of all reactions, 50 µL of Stop Solution was applied to each well, and the plate was immediately measured at 450 nm using a Tecan Infinite 200 Pro MPlex spectrometer. The concentration was measured and compared to a standard curve (0.06 to 18 ng/mL). The results were expressed as mean ± SD (%) vs. control (line 0) for five separate experiments run in duplicate.

### 4.19. NRG1 ELISA Assay

After treatment with ALA and CAR samples, following the manufacturer’s instructions, cell culture supernatants were employed using the NRG1 Rat ELISA Kit (FineTest, Wuhan). In summary, the plate was incubated at 37 °C for 90 min after 100 µL of each sample was added to each well. Following the incubation period, each well’s contents were removed, and Wash Buffer was used twice to wash the wells. The wells were filled with 100 µL of biotin-labelled antibody working solution, and the plate was incubated for 60 min at 37 °C. Following the incubation period, the solution in each well was withdrawn, and Wash Buffer was used three times to wash the wells. After adding 100 µL of SABC Working Solution to each well, the plate was incubated for 30 min at 37 °C. Following five rounds of washing, 90 µL of TMB substrate was added to each well. A plate reader (Infinite 200 Pro MPlex, Tecan, Männedorf, Switzerland) was used to read the plate at 450 nm as soon as 10–20 min had passed after adding 50 µL of Stop Solution to each well. Five independent experiments were conducted in triplicate, and the findings were expressed as mean ± SD (%) versus control (0 line) after the data were gathered and compared to the standard curve (range from 0.156 to 10 ng/mL).

### 4.20. Estrogen Receptor β ELISA Assay

After treatment with ALA and CAR samples, the Rat Estrogen Receptor Beta (ERb) ELISA Kit (Cloud-Clone, Houston, TX, USA) was used on cell lysates, according to the manufacturer’s instructions [58]. In summary, each well was given 100 µL of sample, and the plate was incubated for one hour at 37 °C. After all reactions, 50 µL of Stop Solution was added to each well, and a spectrometer (Infinite 200 Pro MPlex, Tecan) was used to read the plate at 450 nm. After obtaining the concentration and comparing it to a standard curve (0.312 to 20 ng/mL), the mean ± SD (%) vs. control of five independent experiments carried out in triplicate was used to express the findings.

### 4.21. Statistical Analysis

The data acquired using Prism GraphPad statistical software 9.4.1 were processed using one-way analysis of variance (ANOVA) and Bonferroni post hoc tests. A Student’s *t*-test with two tails was adopted to compare the two groups. A two-way ANOVA was conducted to evaluate multiple group comparisons, followed by a two-sided Dunnett post hoc test. The mean ± SD of at least five independent experiments, all performed in triplicate, was used to express all results.

## 5. Conclusions

In conclusion, this study demonstrated how employing M.A.T.R.I.S. technology on ALA and CAR has proven more beneficial for using G.R.M. in a setting mimicking neuropathy in vitro. Our findings demonstrated that using the M.A.T.R.I.S. technology allows us to obtain better results in the intestinal environment with a slower release and prolonged absorption. M.A.T.R.I.S. technology allows peak uptake to occur at a delayed time, thus modulating the release of ALA and CAR over time and thus modulating its effect at the target. Further, ALA and CAR with M.A.T.R.I.S. technology can more efficiently reduce neuropathy than the G.R.M. by modulating the myelinisation mechanism, preventing damage and restoring the altered neurotropism. Ultimately, this study shows that M.A.T.R.I.S. technology can create innovative and safe treatments by providing fresh insights into how it affects absorption mechanisms. Linked to that, future analyses will be carried out to explore the effectiveness of M.A.T.R.I.S. technology in reducing the dosages of substances combined.

## Data Availability

The Laboratory of Physiology stores raw data to ensure permanent retention under a secure system. This study’s data are available from the corresponding author upon reasonable request.

## Abbreviations

The following abbreviations are used in this manuscript:

ALA	α-lipoic acid
AUC	plasma concentration-time curve
CAR	Acetyl-L-carnitine
DSC	Differential Scanning Calorimetry test
ELISA	Enzyme-Linked Immunosorbent Assay
EMA	European Medicines Agency
Erb	beta receptor
FDA	Food and Drug Administration
FT-IR/ATR	Fourier transform infrared spectroscopy /attenuated total reflection
GGF	Glial Growth Factor
G.R.M.	Granular Raw Material
IL-1β	interleukin 1β
M.A.T.R.I.S.	Multiform Administration Timed Release Ingredients System
MPZ	myelin protein-zero
NFkB	Nuclear factor kappa-light-chain-enhancer of activated B cells
NRG1	Neuregulin 1
Papp	apparent permeability coefficient
ROS	radical oxygen species
SEM	scanning electron microscopy
TEER	transepithelial electrical resistance
TNFα	tumour necrosis factor-α
TJs	tight junctions

## References

[1] Zhang A., Jung K., Li A., Liu J., Boyer C. (2019). Recent advances in stimuli-responsive polymer systems for remotely controlled drug release. Prog. Polym. Sci..

[2] Assadpour E., Jafari S.M. (2020). Importance of release and bioavailability studies for nanoencapsulated food ingredients. Release and Bioavailability of Nanoencapsulated Food Ingredients.

[3] Boostani S., Jafari S.M. (2020). Controlled release of nanoencapsulated food ingredients. Release and Bioavailability of Nanoencapsulated Food Ingredients.

[4] McClements D.J. (2014). Nanoparticle-and Microparticle-Based Delivery Systems: Encapsulation, Protection and Release of Active Compounds.

[5] Hillery A., Park K. (2016). Drug Delivery: Fundamentals and Applications.

[6] Adepu S., Ramakrishna S. (2021). Controlled Drug Delivery Systems: Current Status and Future Directions. Molecules.

[7] Nokhodchi A., Raja S., Patel P., Asare-Addo K. (2012). The role of oral controlled release matrix tablets in drug delivery systems. BioImpacts.

[8] Pasotti G., Spalla A., De Zanet E. (2016). International Products and Services IPS SRL. Polyvalent Polymeric Matrix for Modified Release Solid Oral Preparations and Method of Preparation Thereof. US Patent.

[9] Gorąca A., Huk-Kolega H., Piechota A., Kleniewska P., Ciejka E., Skibska B. (2011). Lipoic acid—Biological activity and therapeutic potential. Pharmacol. Rep..

[10] Mayr J.A., Feichtinger R.G., Tort F., Ribes A., Sperl W. (2014). Lipoic acid biosynthesis defects. J. Inherit. Metab. Dis..

[11] Morikawa T., Yasuno R., Wada H. (2001). Do mammalian cells synthesize lipoic acid?: Identification of a mouse cDNA encoding a lipoic acid synthase located in mitochondria. FEBS.

[12] Zehnpfennig B., Wiriyasermkul P., Carlson D.A., Quick M. (2001). Interaction of α-lipoic acid with the human Na^+^/multivitamin transporter (hSMVT). J. Biol. Chem..

[13] Keith D.J., Butler J.A., Bemer B., Dixon B., Johnson S., Garrard M., Sudakin D.L., Christensen J.M., Pereira C., Hagen T.M. (2012). Age and gender dependent bioavailability of R-and R, S-α-lipoic acid: A pilot study. Pharmacol. Res..

[14] Mosallaei N., Malaekeh-Nikouei A., Sarraf Shirazi S., Behmadi J., Malaekeh-Nikouei B. (2024). A comprehensive review on alpha-lipoic acid delivery by nanoparticles. BioImpacts.

[15] Durazzo A., Lucarini M., Nazhand A., Souto S.B., Silva A.M., Severino P., Souto E.B., Santini A. (2020). The Nutraceutical Value of Carnitine and Its Use in Dietary Supplements. Molecules.

[16] Lopez-Maldonado A., Pastoriza S., Rufián-Henares J.Á. (2021). Assessing the antioxidant and metabolic effect of an alpha-lipoic acid and acetyl-L-carnitine nutraceutical. Curr. Res. Food. Sci..

[17] Alhasaniah A.H. (2023). l-carnitine: Nutrition, pathology, and health benefits. Saudi. J. Biol. Sci..

[18] Mignini F., Nasuti C., Gioventu G., Napolioni P.D., Martino P.D. (2012). Human Bioavailability and Pharmacokinetic Profile of Different Formulations Delivering Alpha Lipoic Acid. J. Clin. Cell Immun..

[19] Amenta F., Traini E., Tomassoni D., Mignini F. (2008). Pharmacokinetics of different formulations of tioctic (alpha-lipoic) acid in healthy volunteers. Clin. Exp. Hypertens..

[20] Acetyl-L-Carnitine 500, mg. https://www.protocolforlife.com/wp-content/uploads/2019/06/P0076-Acetyl-L-Carnitine-500-mg.pdf.

[21] El Barky A.R., Hussein S.A., Mohamed T.M. (2017). The potent antioxidant alpha lipoic acid. J. Plant Chem. Ecophysiol..

[22] Maglione E., Marrese C., Migliaro E., Marcuccio F., Panico C., Salvati C., Citro G., Quercio M., Roncagliolo F., Torello C. (2015). Increasing bioavailability of (R)-alpha-lipoic acid to boost antioxidant activity in the treatment of neuropathic pain. Acta Bio-Medica Atenei Parmensis.

[23] Ferreira G.C., McKenna M.C. (2017). L-carnitine and acetyl-L-carnitine roles and neuroprotection in developing brain. Neurochem. Res..

[24] Superti F., Russo R. (2024). Alpha-Lipoic Acid: Biological Mechanisms and Health Benefits. Antioxidants.

[25] Dąbrowska M., Starek M. (2014). Analytical approaches to determination of carnitine in biological materials, foods and dietary supplements. Food Chem..

[26] Brufani M., Figliola R. (2014). (R)-α-lipoic acid oral liquid formulation: Pharmacokinetic parameters and therapeutic efficacy. Acta Bio-Medica Atenei Parmensis.

[27] Salehi B., Berkay Yılmaz Y., Antika G., Boyunegmez Tumer T., Fawzi Mahomoodally M., Lobine D., Akram M., Riaz M., Capanoglu E., Sharopov F. (2019). Insights on the Use of α-Lipoic Acid for Therapeutic Purposes. Biomolecules.

[28] Evans A.M., Fornasini G. (2003). Pharmacokinetics of L-carnitine. Clin. Pharmacokinet..

[29] Cassanego G., Rodrigues P., De Freitas Bauermann L., Trevisan G. (2022). Evaluation of the analgesic effect of a-lipoic acid in treating pain disorders: A systematic review and meta-analysis of randomized controlled trials. Pharmacol. Res..

[30] Rochette L., Ghibu S., Richard C., Zeller M., Cottin Y., Vergely C. (2013). Direct and indirect antioxidant properties of alpha-lipoic acid and therapeutic potential. Mol. Nutr. Food Res..

[31] Di Stefano G., Di Lionardo A., Galosi E., Truini A., Cruccu G. (2019). Acetyl-L-carnitine in painful peripheral neuropathy: A systematic review. J. Pain Res..

[32] Veronese N., Sergi G., Stubbs B., Bourdel-Marchasson I., Tessier D., Sieber C., Strandberg T., Gillain S., Barbagallo M., Crepaldi G. (2017). Effect of acetyl-l-carnitine in the treatment of diabetic peripheral neuropathy: A systematic review and meta-analysis. Eur. Geriatr. Med..

[33] Boerboom A., Dion V., Chariot A., Franzen R. (2017). Molecular mechanisms involved in schwann cell plasticity. Front. Mol. Neurosci..

[34] Pan P., Dobrowsky R.T. (2014). Differential expression of neuregulin-1 isoforms and downregulation of erbin are associated with Erb B2 receptor activation in diabetic peripheral neuropathy. Acta Neuropathol. Commun..

[35] Song W., Jiang W., Wang C., Xie J., Liang X., Sun Y., Gong L., Liu W., Qu L. (2019). Jinmaitong, a traditional Chinese compound prescription, ameliorates the streptozocin-induced diabetic peripheral neuropathy rats by increasing sciatic nerve IGF-1 and IGF-1R expression. Front. Pharmacol..

[36] Song W., Sun Y., Liang X.C., Zhang Q., Xie J., Wang C., Liu W. (2021). Jinmaitong ameliorates diabetes-induced peripheral neuropathy in rats through Wnt/β-catenin signaling pathway. J. Ethnopharmacol..

[37] Molinari C., Morsanuto V., Ghirlanda S., Ruga S., Notte F., Gaetano L., Uberti F. (2019). Role of combined lipoic acid and vitamin D3 on astrocytes as a way to prevent brain ageing by induced oxidative stress and iron accumulation. Oxidative Med. Cell. Longev..

[38] Samuel H.S., Makong Ekpan F. (2024). The use of Scanning Electron Microscopy SEM for Medical Application: A Mini Review. Eurasian J. Sci. Technol..

[39] Hadayanti F. (2020). Application of SEM: Review. Int. J. Appl. Sci. Engenearing Rev..

[40] Klang V., Valenta C., Matsko N.B. (2013). Electron Microscopy of Pharmaceutical System. Micron.

[41] Lea T. (2015). Caco-2 cell line. The Impact of Food Bioactives on Health: In Vitro and Ex Vivo Models.

[42] DiMarco R.L., Hunt D.R., Dewi R.E., Heilshorn S.C. (2017). Improvement of paracellular transport in the Caco-2 drug screening model using protein-engineered substrates. Biomaterials.

[43] Galla R., Grisenti P., Farghali M., Saccuman L., Ferraboschi P., Uberti F. (2021). Ovotransferrin Supplementation Improves the Iron Absorption: An In Vitro Gastro-Intestinal Model. Biomedicines.

[44] Morsanuto V., Galla R., Molinari C., Uberti F. (2020). A New Palmitoylethanolamide Form Combined with Antioxidant Molecules to Improve Its Effectivess on Neuronal Aging. Brain Sci..

[45] Rayner M.L.D., Laranjeira S., Evans R.E., Shipley R.J., Healy J., Phillips J.B. (2018). Developing an In Vitro Model to Screen Drugs for Nerve Regeneration. Anatom Rec..

[46] Chua P., Lim W.K. (2021). Optimisation of a PC12 cell-based in vitro stroke model for screening neuroprotective agents. Sci. Rep..

[47] Park S.E., Ahn J., Jeong H.E., Youn I., Hu D., Chung S. (2021). A three-dimensional in vitro model of the peripheral nervous system. NPG Asia Mater..

[48] Yuan L., Xu H., Guo R., Lu T., Li X. (2021). Long non-coding RNA ZFAS1 alleviates bupivacaine-induced neurotoxicity by regulating the miR-421/zinc finger protein564 (ZNF564) axis. Bioengineered.

[49] Uberti F., Trotta F., Pagliaro P., Bisericaru D.M., Cavalli R., Ferrari S., Penna C., Matencio A. (2023). Developing New Cyclodextrin-Based Nanosponges Complexes to Improve Vitamin D Absorption in an In Vitro Study. Int. J. Mol. Sci..

[50] Christides T., Wray D., McBride R., Fairweather R., Sharp P. (2015). Iron bioavailability from commercially available iron supplements. Eur. J. Nutr..

[51] Fanzaga M., Bollati C., Ranaldi G., Sucato S., Fustinoni S., Roda G., Lammi C. (2023). Bioavailability Assessment of an Iron Formulation Using Differentiated Human Intestinal Caco-2 Cells. Foods.

[52] Mulè S., Rosso G., Botta M., Brovero A., Ferrari S., Galla R., Molinari C., Uberti F. (2024). Design of Mixed Medicinal Plants, Rich in Polyphenols, Vitamins B, and Palmitoylethanolamide-Based Supplement to Help Reduce Nerve Pain: A Preclinical Study. Int. J. Mol. Sci..

[53] Uberti F., Morsanuto V., Ghirlanda S., Molinari C. (2017). Iron Absorption from Three Commercially Available Supplements in Gastrointestinal Cell Lines. Nutrients.

[54] Uberti F., Ruga S., Farghali M., Galla R., Molinari C. (2023). A Combination of α-Lipoic Acid (ALA) and Palmitoylethanolamide (PEA) Blocks Endotoxin-Induced Oxidative Stress and Cytokine Storm: A Possible Intervention for COVID-19. J. Diet. Suppl..

[55] Hamza A.A., Heeba G.H., Elwy H.M., Murali C., El-Awady R., Amin A. (2018). Molecular characterization of the grape seeds extract’s effect against chemically induced liver cancer: In vivo and in vitro analyses. Sci. Rep..

[56] Tomellini E., Lagadec C., Polakowska R., Le Bourhis X. (2014). Role of p75 neurotrophin receptor in stem cell biology: More than just a marker. Cell. Mol. Life Sci..

[57] Ruga S., Galla R., Ferrari S., Invernizzi M., Uberti F. (2023). Novel Approach to the Treatment of Neuropathic Pain Using a Combination with Palmitoylethanolamide and *Equisetum arvense* L. in an In Vitro Study. Int. J. Mol. Sci..

[58] Rzemieniec J., Litwa E., Wnuk A., Lason W., Krzeptowski W., Kajta M. (2016). Selective Aryl Hydrocarbon Receptor Modulator 3,3′-Diindolylmethane Impairs AhR and ARNT Signaling and Protects Mouse Neuronal Cells Against Hypoxia. Mol. Neurobiol..

